# ADAM-like Decysin-1 (ADAMDEC1) is a positive regulator of Epithelial Defense Against Cancer (EDAC) that promotes apical extrusion of RasV12-transformed cells

**DOI:** 10.1038/s41598-018-27469-z

**Published:** 2018-06-25

**Authors:** Yuta Yako, Takashi Hayashi, Yasuto Takeuchi, Kojiro Ishibashi, Nobuhiro Kasai, Nanami Sato, Keisuke Kuromiya, Susumu Ishikawa, Yasuyuki Fujita

**Affiliations:** 1 Division of Molecular Oncology, Institute for Genetic Medicine, Sapporo, 060-0815 Japan; 2 Graduate School of Chemical Sciences and Engineering, Hokkaido University, Sapporo, 060-0815 Japan; 3 Biomedical Technology Research Center, Tokushima Research Institute, Otsuka Pharmaceutical Co., Ltd., Tokushima, 771-0192 Japan

## Abstract

Recent studies have revealed that newly emerging transformed cells are often eliminated from epithelia via cell competition with the surrounding normal epithelial cells. However, it remains unknown whether and how soluble factors are involved in this cancer preventive phenomenon. By performing stable isotope labeling with amino acids in cell culture (SILAC)-based quantitative mass spectrometric analyses, we have identified ADAM-like Decysin-1 (ADAMDEC1) as a soluble protein whose expression is upregulated in the mix culture of normal and RasV12-transformed epithelial cells. Expression of ADAMDEC1 is elevated in normal epithelial cells co-cultured with RasV12 cells. Knockdown of ADAMDEC1 in the surrounding normal cells substantially suppresses apical extrusion of RasV12 cells, suggesting that ADAMDEC1 secreted by normal cells positively regulate the elimination of the neighboring transformed cells. In addition, we show that the metalloproteinase activity of ADAMDEC1 is dispensable for the regulation of apical extrusion. Furthermore, ADAMDEC1 facilitates the accumulation of filamin, a crucial regulator of Epithelial Defense Against Cancer (EDAC), in normal cells at the interface with RasV12 cells. This is the first report demonstrating that an epithelial intrinsic soluble factor is involved in cell competition in mammals.

## Introduction

At the initial step of carcinogenesis, transformation occurs in single cells within epithelial layers. Recent studies have revealed that the newly emerging transformed cells and the surrounding normal epithelial cells often compete with each other for survival and space, a phenomenon called cell competition; the loser cells are eliminated from the tissues, while the winner cells occupy the vacant spaces^1–10^. For example, when RasV12-transformed cells are surrounded by normal epithelial cells, transformed cells are apically eliminated and leave the epithelial tissues^11,12^. During this potentially cancer preventive process, cytoskeletal proteins filamin and vimentin are accumulated in normal cells at the interface with the neighboring transformed cells and actively eliminate the latter cells by generating contractile forces^13^. In addition, accumulation of filamin induces various non-cell-autonomous changes in the neighboring transformed cells such as altered metabolisms, enhanced endocytosis, and reorganization of cytoskeletons, which also positively regulate elimination of transformed cells^12,14,15^. These data imply that normal epithelia display anti-tumor activity that does not involve immune cells, a process termed Epithelial Defense Against Cancer (EDAC)^13^. Several lines of evidence indicate that direct cell-cell interactions between normal and transformed cells trigger cell competition. In *Drosophila*, however, the soluble factor dSparc is transcriptionally upregulated in loser cells during cell competition and protects these cells from apoptotic cell death^16^. In addition, other soluble factors such as Toll-like receptor ligands and Upd3 can also regulate cell competition^17,18^. Moreover, at the early stage of embryogenesis in mice, it has been suggested that soluble factor(s) might be involved in selection of developmentally optimal cells^19^. But, it still remains elusive whether and how soluble factors play a role in cell competition, especially in mammals.

ADAM-like Decysin-1 (ADAMDEC1), a member of the ADAM family, is a secreted type of metalloprotease^20,21^. ADAMDEC1 contains a signal peptide, a prodomain, a catalytic domain, and an incomplete disintegrin domain, but lacks a typical transmembrane domain and a cytoplasmic tail^22^. Moreover, ADAMDEC1 contains the ADAM consensus sequence for the proteolytic activity and exhibits a metalloproteinase activity for α2-macroglobulin (α2M) *in vitro*^20^. However, its physiological function remains largely unknown.

In this study, we demonstrate that normal epithelial cells secrete ADAMDEC1, upon interaction with RasV12-transformed cells, which facilitates apical extrusion of the transformed cells.

## Results

### ADAMDEC1 is identified by SILAC-based analyses as a soluble protein enriched in the co-culture of normal and RasV12-transformed epithelial cells

**Figure 1:**
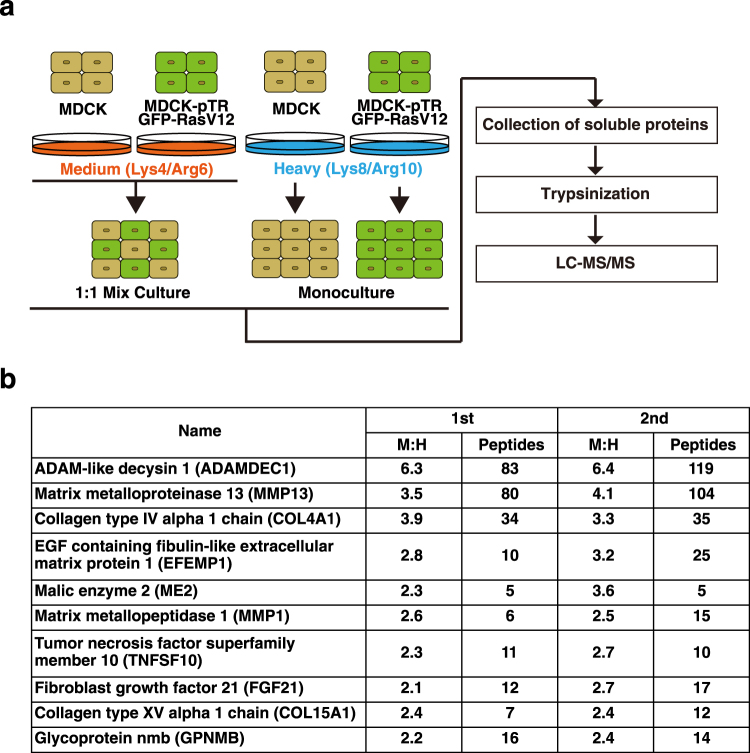
SILAC-based analyses for soluble proteins enriched in the co-culture of normal and RasV12-transformed epithelial cells. (**a**) A scheme for the SILAC-based analyses. MDCK and MDCK-pTR GFP-RasV12 cells were labeled with medium (Lys4/Arg6) or heavy (Lys8/Arg10) lysine and arginine. Medium-labeled normal MDCK cells were mixed with medium-labeled RasV12 cells at a ratio of 1:1 (1:1 Mix Culture), whereas heavy-labeled MDCK or RasV12 cells were cultured separately (Monoculture). At 24 h after tetracycline addition, the culture media were collected, followed by trypsin digestion. Peptides were analyzed by quantitative mass spectrometry using LC-MS/MS. (**b**) Top ten identified proteins whose expression is significantly upregulated in the mix-culture of normal and RasV12 cells. The M:H ratio represents the overall average ratio of the signal intensities of mass spectra for medium-labeled peptides relative to those for heavy-labeled peptides.

**Figure 2:**
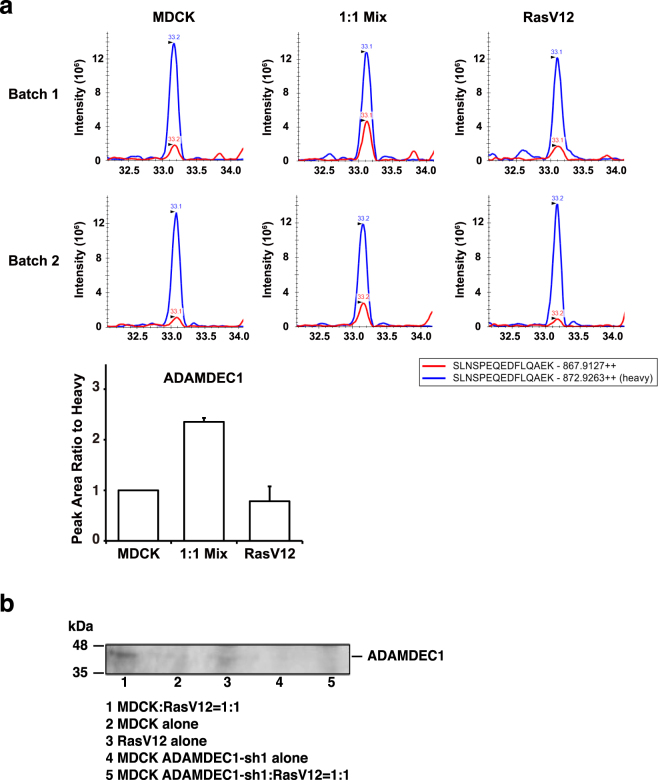
Expression of ADAMDEC1 is increased in the co-culture of normal and RasV12-transfoemed cells. (**a**) MS1 full-scan filtering analysis of ADAMDEC1 in 1:1 co-culture or mono-culture of normal and RasV12-transformed cells. MS1 ion chromatograms of ADAMDEC1 peptide (SLNSPEQEDFLQAEK, in red) or its corresponding heavy internal standard (SLNSPEQEDF[^13^C9,^15^N]LQAEK, in blue) were extracted using skyline software. The level of ADAMDEC1 was normalized to heavy internal standard peak area. Values are expressed as ratios relative to MDCK. Data are mean ± SD from two independent experiments. (**b**) Analysis of ADAMDEC1 expression in conditioned media by western blotting. Conditioned media from the indicated culture conditions were examined by western blotting with anti-human ADAMDEC1 antibody.

To analyze cell competition between normal and RasV12-transformed cells, we have established Madin-Darby canine kidney (MDCK) epithelial cells that stably express RasV12 in a tetracycline-inducible manner^11^. Using this cell line, we have demonstrated that when RasV12-transformed cells are surrounded by normal epithelial cells, the transformed cells are apically extruded from the epithelial monolayer^11^. To identify soluble proteins that are involved in this process, we performed stable isotope labeling with amino acids in cell culture (SILAC)-based quantitative mass spectrometric analyses^23,24^. Two types of isotope-labeled lysine and arginine were used for cell labeling: medium (Lys4, Arg6) and heavy (Lys8, Arg10). Medium-labeled normal MDCK and RasV12 cells were co-cultured, whereas heavy-labeled MDCK and RasV12 cells were cultured separately (Fig. 1a). The culture media were collected from each condition, and those from the monoculture of normal and RasV12 cells were combined, followed by trypsin digestion. Thereafter, the amounts of medium- and heavy-labeled peptides were compared by liquid chromatography-tandem mass spectrometry (LC-MS/MS). The relative protein abundance ratio (M:H ratio) was calculated based on the signal intensities of mass spectra for medium-labeled peptides relative to those for heavy-labeled peptides, which represents the difference between the two culture states. We identified 14 proteins that presented >2 M:H ratio in two independent analyses, and ADAMDEC1 had the highest M:H ratio (Figs 1b and S1a,b). The non-cell-autonomous upregulation of ADAMDEC1 was also confirmed at the protein level by MS1 full-scan filtering and western blotting (Fig. 2a,b). These results indicate that ADAMDEC1 is abundantly secreted from the co-culture of normal and RasV12 cells, compared with their monocultures.

### ADAMDEC1 secreted by normal epithelial cells plays a positive role in apical extrusion of RasV12-transformed cells

**Figure 3:**
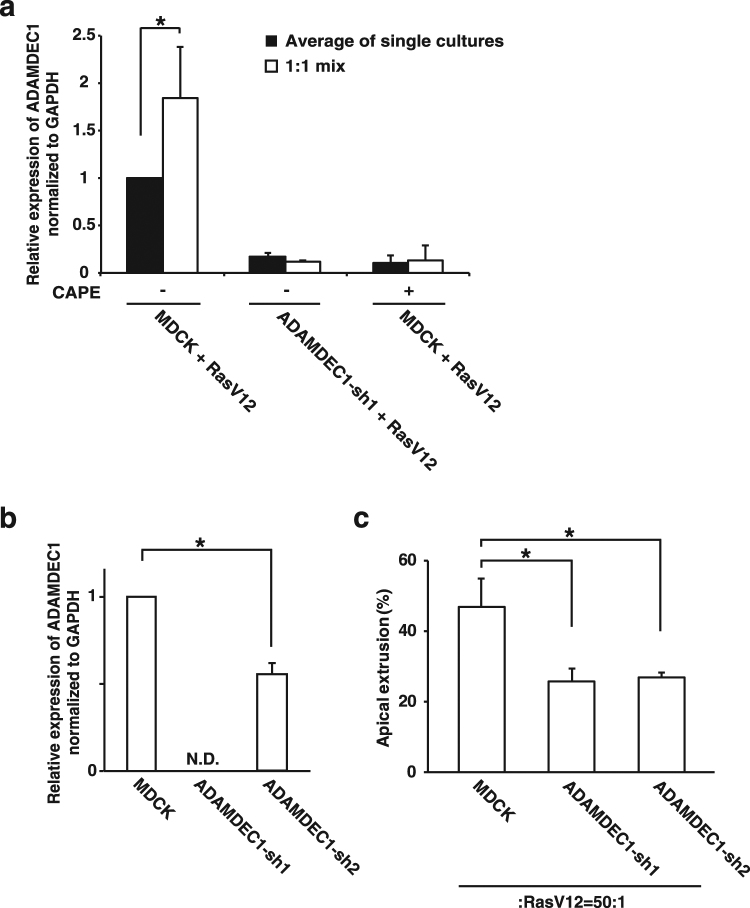
ADAMDEC1 secreted by normal epithelial cells plays a positive role in apical extrusion of RasV12-transformed cells. (**a**) Quantitative real-time PCR analysis of ADAMDEC1 expression. MDCK-pTR GFP-RasV12 cells and MDCK cells or MDCK ADAMDEC1-shRNA1 cells were cultured separately or co-cultured at a ratio of 1:1 in the absence or presence of the NF-κB inhibitor CAPE. The relative mRNA level of ADAMDEC1 was measured by quantitative real-time PCR with GAPDH mRNA as internal control. Average values from single culture of RasV12 cells and MDCK cells (or MDCK ADAMDEC1-shRNA1 cells) are shown as black bars. Values are expressed as ratios relative to average of single cultures of MDCK and RasV12 cells. Data are mean ± SD from eight (left), three (middle), or five (right) independent experiments. ^*^P < 0.05, unpaired Student’s *t*-test. (**b**) Quantitative real-time PCR analyses for ADAMDEC1 expression in MDCK cells stably expressing ADAMDEC1-shRNA1 or -shRNA2. Values are expressed as ratios relative to MDCK. Data are mean ± SD from two independent experiments. ^*^P < 0.05, unpaired Student’s *t*-test. N.D.; not detected. (**c**) Effect of ADAMDEC1-knockdown on apical extrusion of RasV12 cells. MDCK-pTR GFP-RasV12 cells were co-cultured with MDCK cells or MDCK ADAMDEC1-shRNA1 or -shRNA2 cells, and at 24 h after tetracycline addition, apical extrusion of RasV12 cells was quantified. Data are mean ± SD from three independent experiments. ^*^P < 0.005, two-tailed Student’s *t*-test.

Next, we examined whether ADAMDEC1 expression is elevated in normal or RasV12-transformed epithelial cells under the co-culture condition by performing quantitative real-time PCR. The expression level of ADAMDEC1 was substantially increased in the co-culture of normal and RasV12 cells, compared with that in single cultures of normal and RasV12 cells (Fig. 3a). This result suggests that the expression of ADAMDEC1 is transcriptionally controlled in a non-cell-autonomous fashion, though we cannot exclude the possibility that it is also regulated at the post-transcriptional level. Previous studies have demonstrated that various non-cell-autonomous changes occur in normal cells, upon interaction with transformed cells, which induce EDAC^13,25^. To examine whether ADAMDEC1 secreted by normal cells is also involved in EDAC, we established MDCK cells stably expressing ADAMDEC1-shRNA (Fig. 3b). Knockdown of ADAMDEC1 in normal cells profoundly suppressed ADAMDEC1 expression at both mRNA and protein levels in the co-culture of normal and RasV12 cells (Figs 2b, 3a and S2a,b), suggesting that ADAMDEC1 expression is upregulated in normal cells under the co-culture condition. Furthermore, when RasV12 cells were surrounded by ADAMDEC1-knockdown cells, apical extrusion of the transformed cells was significantly suppressed (Fig. 3c). Collectively, these results suggest that ADAMDEC1 secreted by normal epithelial cells promotes apical extrusion of the neighboring RasV12-transformed cells.

### The NF-κB pathway is involved in ADAMDEC1 expression and apical extrusion

**Figure 4:**
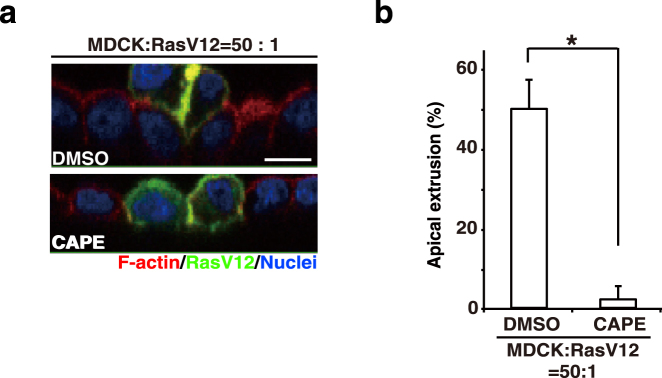
The NF-κB inhibitor CAPE suppresses apical extrusion of RasV12-transformed cells. (**a**,**b**) Effect of CAPE on apical extrusion of RasV12-transformed cells. MDCK and MDCK-pTR GFP-RasV12 cells were co-cultured in the absence or presence of CAPE. Cells were fixed at 24 h after tetracycline addition and stained with Alexa-Fluor-568-phalloidin (red) and Hoechst (blue). (**a**) Confocal microscopic immunofluorescence images of xz sections. Scale bars, 10 μm. (**b**) Quantification of apical extrusion of RasV12 cells. Data are mean ± SD from three independent experiments. ^*^P < 0.005, two-tailed Student’s *t*-test.

According to the analyses with UCSC Genome Browser, the promoter regions of *ADAMDEC1* contain regulatory sequences for various transcriptional factors, among which NF-κB, EBF1, and CTCF show high confidence (Fig. S3a). As a previous study reported the involvement of the NF-κB pathway in cell competition in *Drosophila*^18^, we further studied a role for NF-κB in the regulation of ADAMDEC1. Treatment with the NF-κB inhibitor CAPE profoundly suppressed expression of ADAMDEC1 (Fig. 3a). Furthermore, the treatment of CAPE markedly decreased the frequency of apical extrusion of RasV12-transformed cells (Fig. 4a,b). However, we did not obtain evidence indicating that the NF-κB pathway was activated in normal cells surrounding RasV12 cells (Fig. S3b). Collectively, these data suggest that the basal activity of the NF-κB pathway is required for ADAMDEC1 expression and apical extrusion, but that these processes are also regulated by an additional signaling pathway(s) that is modulated between normal and RasV12 cells in a non-cell-autonomous fashion.

### The metalloproteinase activity of ADAMDEC1 is dispensable for the regulation of apical extrusion

**Figure 5:**
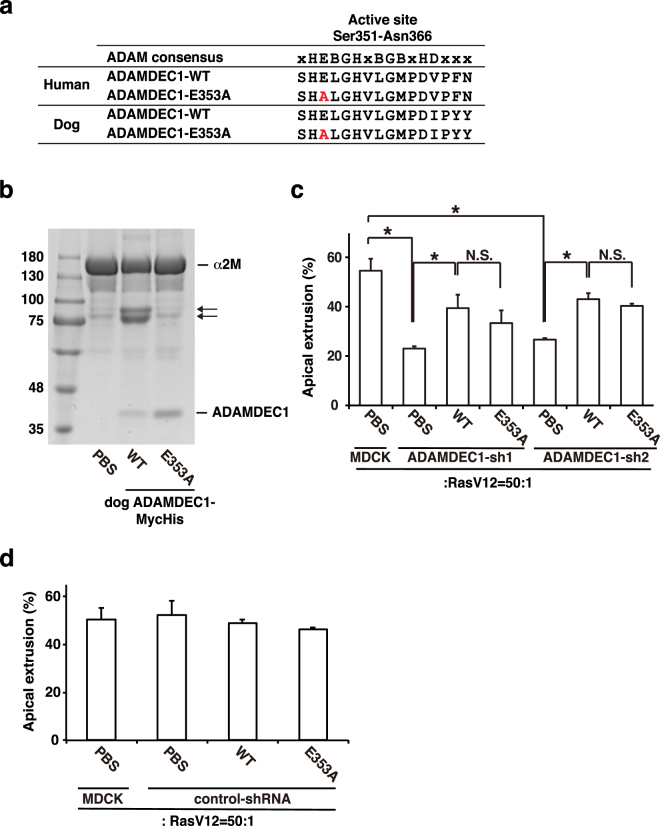
The metalloproteinase activity of ADAMDEC1 is dispensable for the regulation of apical extrusion. (**a**) Amino acid sequence alignment of the predicted active site (Ser351-Asn366) in human and dog ADAMDEC1. Red letters indicate the E353A substitution. X: less conserved residue; B: bulky hydrophobic residue. (**b**) *In vitro* proteolytic activity assay of ADAMDEC1-WT and -E353A. The substrate α2 M protein was incubated with ADAMDEC1-WT or -E353A, followed by SDS-PAGE and Coomassie Brilliant Blue protein staining. The arrows indicate cleaved α2 M. (**c**,**d**) Effect of addition of ADAMDEC1-WT or -E353A on apical extrusion of RasV12-transformed cells surrounded by ADAMDEC1-knockdown or control-shRNA-expressing cells. MDCK-pTR GFP-RasV12 cells were cultured with MDCK, MDCK ADAMDEC1-shRNA1, -shRNA2 (**c**) or control-shRNA (**d**) cells in the absence or presence of ADAMDEC1-WT or -E353A recombinant proteins, and apical extrusion of RasV12 cells was quantified at 24 h after tetracycline addition. Data are mean ± SD from two independent experiments. ^*^P < 0.05, unpaired Student’s *t*-test. N.S.; not significant.

Human ADAMDEC1 has metalloprotease activity, and the glutamate 353 (E353) residue in the ADAM consensus sequence is important for this activity^20^. Canine ADAMDEC1 also has the ADAM consensus sequence representing the protease active site, in which E353 is conserved (Fig. 5a). We purified canine wild-type (WT) and glutamate mutant (E353A) ADAMDEC1 recombinant proteins and analyzed their protease activities *in vitro*. By examining the cleavage of human α2 M, we demonstrated that ADAMDEC1-WT possessed proteolytic activity, but ADAMDEC1-E353A did not (Fig. 5b), indicating the evolutionarily conserved protease activity of canine ADAMDEC1. Next, we examined whether addition of ADAMDEC1-WT or -E353A to the media rescues the inhibitory effect of ADAMDEC1-knockdown on apical extrusion of RasV12-transformed cells. ADAMDEC1-WT restored the frequency of apical extrusion of RasV12 cells surrounded by ADAMDEC1-knockdown cells (Fig. 5c). In addition, ADAMDEC1-E353A rescued this phenotype to a comparable extent (Fig. 5c). ADAMDEC1-WT or -E353A did not significantly affect apical extrusion of RasV12 cells surrounded by control-shRNA-expressing cells (Fig. 5d). Collectively, these data indicate that ADAMDEC1 facilitates apical extrusion independently of its metalloprotease activity.

### ADAMDEC1 acts upstream of filamin in EDAC

**Figure 6:**
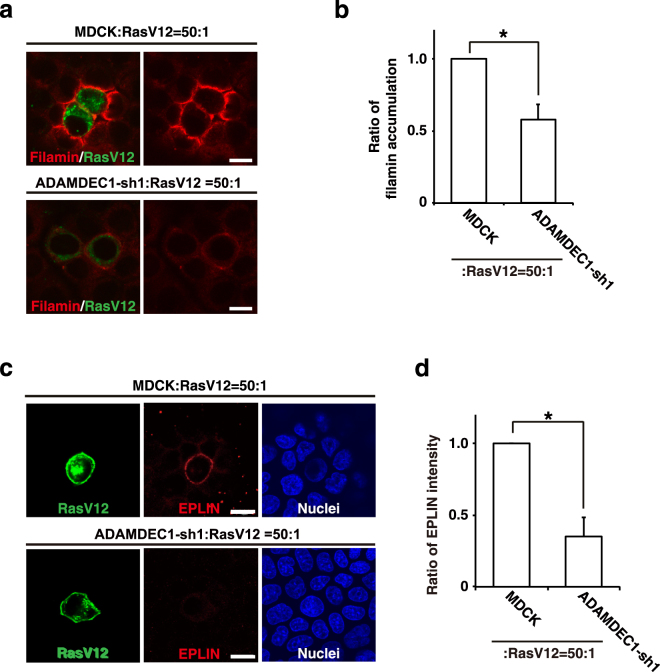
Knockdown of ADAMDEC1 suppresses filamin accumulation in normal cells and EPLIN accumulation in RasV12-transformed cells at their interface. (**a**,**b**) MDCK-pTR GFP-RasV12 cells were co-cultured with MDCK or MDCK ADAMDEC1-shRNA1 cells. Cells were fixed at 16 h after tetracycline addition and stained with anti-filamin antibody (red). (**a**) Confocal microscopic immunofluorescence images of filamin. (**b**) Quantification of filamin accumulation. Data are mean ± SD from five independent experiments. (**c**,**d**) MDCK-pTR GFP-RasV12 cells were co-cultured with MDCK or MDCK ADAMDEC1-shRNA1 cells. Cells were fixed at 16 h after tetracycline addition and stained with anti-EPLIN antibody (red) and Hoechst (blue). (**c**) Confocal microscopic immunofluorescence images of EPLIN. (**a**,**c**) Scale bars, 10 μm. (**d**) Quantification of EPLIN accumulation. Data are mean ± SD from three independent experiments. Values are expressed as ratios relative to MDCK: RasV12 = 50:1. ^*^P < 0.05, two-tailed Student’s *t*-test.

Filamin, a crucial regulator of EDAC, accumulates in normal epithelial cells at the interface with the neighboring RasV12-transformed cells and thereby positively regulates apical elimination of transformed cells (Fig. 6a)^13^. When RasV12 cells were surrounded by ADAMDEC1-knockdown cells, accumulation of filamin was substantially diminished (Figs 6a,b and S4), suggesting that ADAMDEC1 regulates filamin accumulation and thus acts as an upstream regulator for the eradication of transformed cells from epithelia. A previous study showed that Epithelial protein lost in neoplasm (EPLIN) is accumulated in RasV12 cells when they are surrounded by normal cells, which regulate filamin accumulation in normal cells, and *vice versa*^14^. We found that accumulation of EPLIN was substantially diminished in RasV12 cells that were surrounded by ADAMDEC1-knockdown cells (Fig. 6c,d).

## Discussion

In *Drosophila*, soluble factors have been shown to play a role in cell competition^16–18^. For example, dSparc, the *Drosophila* homolog of the SPARC/Osteonectin protein family, is transcriptionally upregulated in loser cells at the early stage of cell competition and protects these cells from apoptosis by inhibiting caspase activation^16^. In addition, a previous study suggested the presence of a soluble factor(s) that positively regulates cell competition during embryonic development in mice, though identity of the soluble factor(s) remains unraveled^19^. In this study, we demonstrate that the soluble protein ADAMDEC1 plays a positive role in apical extrusion of RasV12-transformed cells from the normal epithelial layer; this is the first report demonstrating that an epithelial intrinsic soluble factor is involved in cell competition in mammals. Our preliminary data show that conditioned media from the co-culture of normal and RasV12-transformed cells do not induce apical extrusion of RasV12 cells cultured alone. Moreover, cell competition generally occurs between directly contacting cells at the boundary of two different populations in both *Drosophila* and mammals. Thus, it is plausible that soluble factors alone may be insufficient to trigger cell competition, and direct interactions between loser and winner cells are also required.

Upon interaction with RasV12-transformed cells, normal cells secrete ADAMDEC1 and thereby influences the behavior of themselves in an autocrine manner by inducing filamin accumulation at the interface with the transformed cells. Accumulation of EPLIN is suppressed in RasV12 cells when they are surrounded by ADAMDEC1-knockdown cells. This may be caused by decreased accumulation of filamin in ADAMDEC1-knockdown cells, but it is also possible that ADAMDEC1 directly influences RasV12 cells in a paracrine fashion. Furthermore, a previous study has demonstrated that exogenous sphingosine-1-phosphate (S1P) binds to S1PR on normal cells and thereby promotes apical extrusion of the neighboring RasV12 cells, implying that extrinsic factors from outer environments can influence the outcome of cell competition^25^. In future studies, we would like to examine whether and how endogenous ADAMDEC1 and exogenous S1P co-regulate the competitive interaction between normal and transformed cells.

Using an *in vitro* proteolytic activity assay, we show that the metalloproteinase activity of ADAMDEC1 is not involved in apical extrusion of RasV12 cells, indicating that ADAMDEC1 promotes apical extrusion independently of its protease activity. In addition to proteolysis, ADAM proteins are involved in other cellular processes such as cell adhesion, cell fusion, and regulation of postsynaptic excitation^26,27^. For example, ADAM22 forms a ligand-receptor complex with LGI1, and thereby enhances AMPA receptor-mediated synaptic transmission^28^. Similarly, ADAMDEC1 may bind to its cognate membrane receptor and activate a signaling pathway(s) upstream of filamin accumulation; this possibility needs to be investigated in future studies.

The molecular mechanism that triggers cell competition between normal and RasV12-transformed cells still remains elusive. However, it is likely that normal cells sense certain physical or chemical differences in RasV12 cells and accordingly activate a signaling pathway(s), eventually leading to upregulation of ADAMDEC1. Treatment with the NF-κB inhibitor CAPE profoundly suppresses expression of ADAMDEC; however the NF-κB pathway is not activated in normal cells neighboring RasV12 cells, suggesting that transcription of ADAMDEC1 is regulated by additional signaling pathways in concert with NF-κB. According to analyses with UCSC Genome Browser, the promoter regions of ADAMDEC1 contain transcriptional regulatory sequences for a number of other transcriptional factors. Identification of upstream transcription factors that control ADAMDEC1 expression will help to elucidate unidentified cell competition-specific signaling pathways.

## Materials and Methods

### Antibodies and Materials

Mouse anti-filamin (F6682) antibody was purchased from Sigma-Aldrich. Rabbit anti-p65 (sc-109) and mouse anti-EPLIN (sc-136399) antibodies were from Santa Cruz Biotechnology. Mouse anti-ADAMDEC1 antibody was kindly provided by Drs. Lund and Petersen (Novo Nordisk A/S)^29^. For immunofluorescence, the primary antibodies were diluted in phosphate-buffered saline (PBS) containing 1% bovine serum albumin (BSA) at 1:100. Alexa-Fluor-568- and -647-conjugated secondary antibodies (Life Technologies) were used at 1:200. Alexa-Fluor-568- and -647-conjugated phalloidin (Life Technologies) was used at 1.0 U ml^−1^. Hoechst 33342 (Life Technologies) was used at a dilution of 1:5,000. Stable isotope-labeled amino acids were purchased from Cambridge Isotope Laboratories. The synthetic peptide containing stable isotope labeled phenylalanine (SLNSPEQEDF[^13^C9,^15^N]LQAEK) was obtained from SCRUM Inc.

### Cell Culture

MDCK cells stably expressing GFP-RasV12 in a tetracycline-inducible manner (MDCK-pTR GFP-RasV12) were established and cultured as previously described^11^. To establish MDCK cells stably expressing ADAMDEC1-shRNA1, -shRNA2, and control-shRNA, ADAMDEC1 shRNA oligonucleotides (ADAMDEC1-shRNA1: 5′-GATCCCCGGCATTGGTAGGTATGGAATTCAAGAGATTCCATCCTACCAATGCCTTTTTC-3′ and 5′-TCGAGAAAAGGCATTGGTAGGTATGGAATCTCTTGAATTCCATACCTACCAATGCCGGG-3′, ADAMDEC1-shRNA2: 5′-GATCCCCGGGCCAGACTACACTGAAATTCAAGAGATTTCAGTGTAGTCTGGCCCTTTTTC-3′ and 5′-TCGAGAAAAAGGGCCAGACTACACTGAAATCTCTTGAATTTCAGTGTAGTCTGGCCCGGG-3′) or Luciferase shRNA oligonucleotides (Luciferase-shRNA: GATCCCCTGAAACGATATGGGCTGAATTCAAGAGATTCAGCCCATATCGTTTCATTTTTC-3′ and 5′-TCGAGAAAAATGAAACGATATGGGCTGAATCTCTTGAATTCAGCCCATATCGTTTCAGGG-3′) were cloned into the *Bgl*II and *Xho*I site of pSUPER.neo + gfp (Oligoengine). MDCK cells were then transfected with pSUPER.neo + gfp ADAMDEC1-shRNA1, -shRNA2, or control-shRNA using Lipofectamine 2000 (Invitrogen), followed by antibiotic selection in the medium containing 800 μg ml^−1^ G418 (Life Technologies). To induce the expression of GFP-RasV12, MDCK-pTR GFP-RasV12 cells were treated with 2 μg ml^−1^ tetracycline (Sigma-Aldrich). Where indicated, the NF-κB inhibitor CAPE (25 μM, Santa Cruz Biotechnology) was added together with tetracycline.

### Stable Isotope Labeling with Amino Acids in Cell Culture

For SILAC labeling, cells were maintained in DMEM minus L-Lysine and L-Arginine (Cambridge Isotope Laboratories) supplemented with 10% dialyzed FCS and 1% penicillin/streptomycin (Gibco). Then, 50 μg ml^−1^ of U-^13^C6-^15^N2 -L-lysine (Lys8) and 50 μg ml^−1^ of U-^13^C6-^15^N4-L-arginine (Arg10) were added for “heavy” labeling, whereas 50 μg ml^−1^ of U-^2^H4-L-lysine (Lys4) and 50 μg ml^−1^ of U-^13^C6-L-arginine (Arg6) were added for “medium” labeling. In addition, 200 μg ml^−1^ of L-proline (Sigma-Aldrich) was added to the culture medium to prevent arginine to proline conversion. After two weeks of SILAC labeling, 1.0 × 10^7^ medium-labeled MDCK cells and 1.0 × 10^7^ medium-labeled MDCK-pTR GFP-RasV12 cells were mixed and co-cultured in a 145-mm dish. On the other hand, heavy-labeled MDCK or MDCK-pTR GFP-RasV12 cells were separately cultured in a 145-mm dish. The SILAC labeled cells were cultured without tetracycline for 8–12 h, and then treated with 2 μg ml^−1^ tetracycline for 24 h. During the last 12 h tetracycline treatment, cells were cultured in FCS-free medium. The cell growth speed of normal or RasV12 cells was not grossly affected by co-culture, and the total cell numbers were eventually comparable between the medium and heavy SILAC culture conditions. The conditioned media were then collected and concentrated to 150 μl by ultrafiltration using Amicon Ultra Centrifugal Filters 3 kDa (Millipore) according to manufacture’s instructions.

### SDS-PAGE and In-Gel Trypsin Digestion

The concentrated proteins were separated in SDS-PAGE and stained with Flamingo™ Fluorescent Get Stain (Bio-Rad). Gels were sliced into 16–18 gel fractions, and each gel piece was reduced with 10 mM of DTT at 55 °C for 45 min, and then alkylated with 55 mM iodoacetamide at room temperature in a dark for 30 min, and further incubated with 2 μg ml^−1^ trypsin in 100 mM ammonium bicarbonate at 37 °C for 16 h. After digestion, peptides were recovered and purified with StageTips^30^.

### LC-MS/MS Analysis

In-gel tryptic peptides were analyzed with Q Exactive or Orbitrap Fusion mass spectrometer coupled with the U3000 nano-UHPLC system. Peptide mixtures were loaded onto C18 Acclaim PepMap precolumn (Thermo Fisher Scientific) and separated by reversed-phase chromatography with a packed tip column (75-μm i.d., 12-cm long, 3-μm C18 particles, Nikkyo Technos) using a linear gradient of 4 to 35% acetonitrile in 0.1% formic acid for 108 min with a flow rate of 300 nl/min. The voltage applied to the nano-electrospray source was 1.8 kV, and mass spectra were obtained in a data-dependent acquisition mode. In the Q Exactive, full scan MS spectra were acquired in a range from m/z 350 to 1,600 at resolution 70,000, and the 10 most intense ions were sequentially selected and fragmented in HCD. In the Orbitrap Fusion, full scan MS spectra were acquired in a range from m/z 350 to 1,600 at resolution 240,000, and the most intense ions within a 3-second cycle time were sequentially subjected to HCD fragmentation and detected in ion-trap. Data analysis was performed in Proteome Discoverer 1.4 software with the Mascot search engine. All MS/MS spectra were searched against the Uniprot dog proteome database UP000002254 (downloaded at October 17, 2017). Peptide-spectrum matches (PSMs) were validated using Percolator at a 1% false discovery rate and further filtered using a mascot ion score >20. For SILAC quantification, medium/heavy ratio (M:H) was calculated by Proteome Discoverer 1.4, and proteins that contain more than 5 SILAC peptides were listed. Data are available via ProteomeXChange repository with identifier PXD008209.

### Quantitative analysis of ADAMDEC1 by mass spectrometry

For label-free ADAMDEC1 quantitation using mass spectrometry, FCS-free conditioned media from monoculture of MDCK, monoculture of RasV12-transformed cells, or 1:1 mixed culture of MDCK and RasV12 cells was collected and concentrated as described before. Proteins were separated in SDS-PAGE, and the gels from the corresponding molecular weight region of ADAMDEC1 were excised and subjected to in-gel trypsin digestion. Then, 100 fmol of stable isotope-labeled peptide standard for ADAMDEC1 (SLNSPEQEDF[^13^C9,^15^N]LQAEK) was spiked into each sample and analyzed with the Orbitrap Fusion LC-MS system as described before. Protein identification was performed in proteome discoverer, and MS1 full-scan filtering was performed in Skyline software (version 3.7) by importing the result from proteome discoverer. The m/z of three precursor isotopes and retention time for chromatogram extraction were determined according to the peptide identification information^31^.

### Quantitative Real-Time PCR (qRT-PCR)

For monoculture or 1:1 mixed culture of RasV12 cells with MDCK or MDCK ADAMDEC1-shRNA1 cells, total 2 × 10^7^ cells were seeded on collagen-coated 145-mm dishes. Total RNAs were extracted from isolated cells with TRIzol™ (Thermo Fisher Scientific), purified using RNeasy Mini Kit (QIAGEN), and reverse transcribed using QuantiTect Reverse Transcription Kit (QIAGEN). GeneAce SYBR qPCR Mix (NIPPON GENE) was used to perform qPCR with the StepOnePlus™ system (Thermo Fisher Scientific). In brief, PCR amplification of the targeted fragments was performed with 45 cycles of denaturation at 95 °C (30 s), annealing and extension at 60 °C (30 s). The primer sequences were as fellows. ADAMDEC1: 5′-TGCCGTTCACATTTTGAGAGATAC-3′ and 5′-CCTCACAGCAAGGATTGGAAC-3′; GAPDH: 5′-ATTCTATCCACGGCAAATCC-3′ and 5′-GGACTCCACAACATACTCAG-3′; β-actin: 5′-GGCACCCAGCACAATGAAG-3′ and 5′- ACAGTGAGGCCAGGATGGAG-3′. For data analysis, relative quantification analysis was performed using the comparative CT (2^−∆∆CT^) method. For each sample, mRNA levels of ADAMDEC1 were normalized to the GAPDH mRNA or β-actin mRNA. The details of calculation processes are demonstrated in Table S2. We have measured the amplification efficiency of each qPCR primer and confirmed that the amplification efficiency is comparable between the three primers (90–100%).

### Immunofluorescence

MDCK-pTR GFP-RasV12 cells were mixed with MDCK or MDCK ADAMDECA1-shRNA1 cells at a ratio of 1:50 and cultured on the collagen matrix. Type-I collagen (Cellmatrix Type I-A) was obtained from Nitta Gelatin (Osaka, Japan) and neutralized on ice to a final concentration of 2 mg ml^−1^ according to the manufacturer’s instructions. The mixture of cells was incubated for 8–12 h until they formed an epithelial monolayer, followed by tetracycline treatment for 16 h for analyses of accumulation of filamin or EPLIN or 24 h for those of apical extrusion. Cells were fixed with 4% paraformaldehyde in PBS and permeabilized with 0.5% Triton X-100 in PBS, followed by blocking with 1% BSA in PBS. Primary or secondary antibodies were incubated for 2 h or 1 h, respectively. Immunofluorescence images were analyzed by the Olympus FV1000 system and Olympus FV10-ASW software. Quantification of immunofluorescence intensity of filamin or EPLIN was performed as previously described^13,14^. For quantification of apical extrusion, more than 100 RasV12 cells were analyzed for each experimental condition. RasV12 cells that have been completely detached from the basal matrix are defined as apically extruded cells.

### Western Blotting

FCS-free-conditioned media from the indicated culture conditions were collected as described before. Each conditioned medium was concentrated by Vivaspin 20 (GE healthcare) and Amicon® Ultra 0.5 mL (Merck Millipore Ltd.) from 15 ml to 80 μl according to the manufacturer’s instructions. Concentrated media were subjected to SDS-PAGE analysis, followed by western blotting with anti-ADAMDEC1 monoclonal antibody. Western blotting was performed as previously described^32^. Primary antibody was used at 1:250. Western blotting data were analyzed using ImageQuant LAS4010 (GE healthcare).

### Purification of Dog ADAMDEC1 Recombinant Proteins

For collection of dog ADANDEC1 recombinant proteins, HEK293 cells were transfected with pCA10^+^ dog ADAMDEC1(WT or E353A)-MycHis using polyethylenimine “MAX” (Polyscience) and cultured for 48 h. In the latter 24 h, cells were cultured in FCS-free media. Secreted His-tagged ADAMDEC1 proteins were collected with Ni-NTA agarose (QIAGEN), followed by purification through the PD-10 column using PBS as elution buffer (GE Healthcare). Protein concentration was estimated by SDS-PAGE using BSA as standard control.

### Proteolytic Activity Assay

Proteolytic activity assay was performed as previously described^20^. In brief, 0.9 μg ml^−1^ of human plasma-derived α2 M was incubated with 100 μg ml^−1^ of ADAMDEC1-WT or -E353A proteins for 65 h at 37 °C. The result was visualized by SDS-PAGE, and cleavage of α2 M was observed by appearance of ∼85-kDa fragments.

### Data Analyses

Unpaired or two-tailed Student’s *t*-tests were used to determine *P*-values for statistical analyses. P < 0.05 was considered to be statistically significant.

### Data Availability

The datasets generated and/or analyzed during the current study are available from the corresponding author on reasonable request.

## Electronic supplementary material

Supplementary Fig. 1-5

Supplementary Table 1

Supplementary Table 2

## References

[1] Amoyel M, Bach EA (2014). Cell competition: how to eliminate your neighbours. Development.

[2] Maruyama T, Fujita Y (2017). Cell competition in mammals - novel homeostatic machinery for embryonic development and cancer prevention. Curr Opin Cell Biol.

[3] Vincent JP, Fletcher AG, Baena-Lopez LA (2013). Mechanisms and mechanics of cell competition in epithelia. Nat Rev Mol Cell Biol.

[4] Johnston LA (2009). Competitive interactions between cells: death, growth, and geography. Science.

[5] Wagstaff L, Kolahgar G, Piddini E (2013). Competitive cell interactions in cancer: a cellular tug of war. Trends in Cell Biology.

[6] Morata G, Ballesteros-Arias L (2015). Cell competition, apoptosis and tumour development. Int J Dev Biol.

[7] Di Gregorio A, Bowling S, Rodriguez TA (2016). Cell Competition and Its Role in the Regulation of Cell Fitness from Development to Cancer. Dev Cell.

[8] Claveria C, Torres M (2016). Cell Competition: Mechanisms and Physiological Roles. Annual review of cell and developmental biology.

[9] Merino MM, Levayer R, Moreno E (2016). Survival of the Fittest: Essential Roles of Cell Competition in Development, Aging, and Cancer. Trends Cell Biol.

[10] Baker NE (2017). Mechanisms of cell competition emerging from Drosophila studies. Curr Opin Cell Biol.

[11] Hogan C (2009). Characterization of the interface between normal and transformed epithelial cells. Nat Cell Biol.

[12] Kon S (2017). Cell competition with normal epithelial cells promotes apical extrusion of transformed cells through metabolic changes. Nat Cell Biol.

[13] Kajita M (2014). Filamin acts as a key regulator in epithelial defence against transformed cells. Nature communications.

[14] Ohoka A (2015). EPLIN is a crucial regulator for extrusion of RasV12-transformed cells. J Cell Sci.

[15] Saitoh S (2017). Rab5-regulated endocytosis plays a crucial role in apical extrusion of transformed cells. Proc Natl Acad Sci USA.

[16] Portela M (2010). Drosophila SPARC is a self-protective signal expressed by loser cells during cell competition. Dev Cell.

[17] Kolahgar G (2015). Cell Competition Modifies Adult Stem Cell and Tissue Population Dynamics in a JAK-STAT-Dependent Manner. Dev Cell.

[18] Meyer SN (2014). An ancient defense system eliminates unfit cells from developing tissues during cell competition. Science.

[19] Sancho M (2013). Competitive interactions eliminate unfit embryonic stem cells at the onset of differentiation. Dev Cell.

[20] Lund J (2013). ADAMDEC1 is a metzincin metalloprotease with dampened proteolytic activity. The Journal of biological chemistry.

[21] Balakrishnan L (2014). Proteomic analysis of human osteoarthritis synovial fluid. Clin Proteomics.

[22] Bates EE, Fridman WH, Mueller CG (2002). The ADAMDEC1 (decysin) gene structure: evolution by duplication in a metalloprotease gene cluster on chromosome 8p12. Immunogenetics.

[23] Mann M (2006). Functional and quantitative proteomics using SILAC. Nat Rev Mol Cell Biol.

[24] Jorgensen C (2009). Cell-specific information processing in segregating populations of Eph receptor ephrin-expressing cells. Science.

[25] Yamamoto S (2016). A role of the sphingosine-1-phosphate (S1P)-S1P receptor 2 pathway in epithelial defense against cancer (EDAC). Mol Biol Cell.

[26] Fukata Y, Yokoi N, Miyazaki Y, Fukata M (2017). The LGI1-ADAM22 protein complex in synaptic transmission and synaptic disorders. Neurosci Res.

[27] Novak U (2004). ADAM proteins in the brain. J Clin Neurosci.

[28] Fukata Y (2006). Epilepsy-related ligand/receptor complex LGI1 and ADAM22 regulate synaptic transmission. Science.

[29] Lund J (2018). Monoclonal antibodies targeting the disintegrin-like domain of ADAMDEC1 modulates the proteolytic activity and enables quantification of ADAMDEC1 protein in human plasma. MAbs.

[30] Rappsilber J, Ishihama Y, Mann M (2003). Stop and go extraction tips for matrix-assisted laser desorption/ionization, nanoelectrospray, and LC/MS sample pretreatment in proteomics. Anal Chem.

[31] Schilling B (2012). Platform-independent and label-free quantitation of proteomic data using MS1 extracted ion chromatograms in skyline: application to protein acetylation and phosphorylation. Mol Cell Proteomics.

[32] Hogan C (2004). Rap1 regulates the formation of E-cadherin-based cell-cell contacts. Mol Cell Biol.

