# Supplementary material for: ADAM-like Decysin-1 (ADAMDEC1) is a positive regulator of Epithelial Defense Against Cancer (EDAC) that promotes apical extrusion of RasV12-transformed cells
Source: Sci Rep. 2018 Jun 25;8:9639. doi: 10.1038/s41598-018-27469-z (PMC6018119; doi:10.1038/s41598-018-27469-z)
Supplement: Supplementary file 1 — Supplementary Fig. 1-5 [file 41598_2018_27469_MOESM1_ESM.pdf]

Supplementary Information

ADAM-like Decysin-1 (ADAMDEC1) is a positive regulator of Epithelial Defense Against Cancer (EDAC) that promotes apical extrusion of RasV12-transformed cells

Yuta Yako, Takashi Hayashi, Yasuto Takeuchi, Kojiro Ishibashi, Nobuhiro Kasai, Nanami Sato, Keisuke Kuromiya, Susumu Ishikawa and Yasuyuki Fujita

Supplementary Figure S1-5

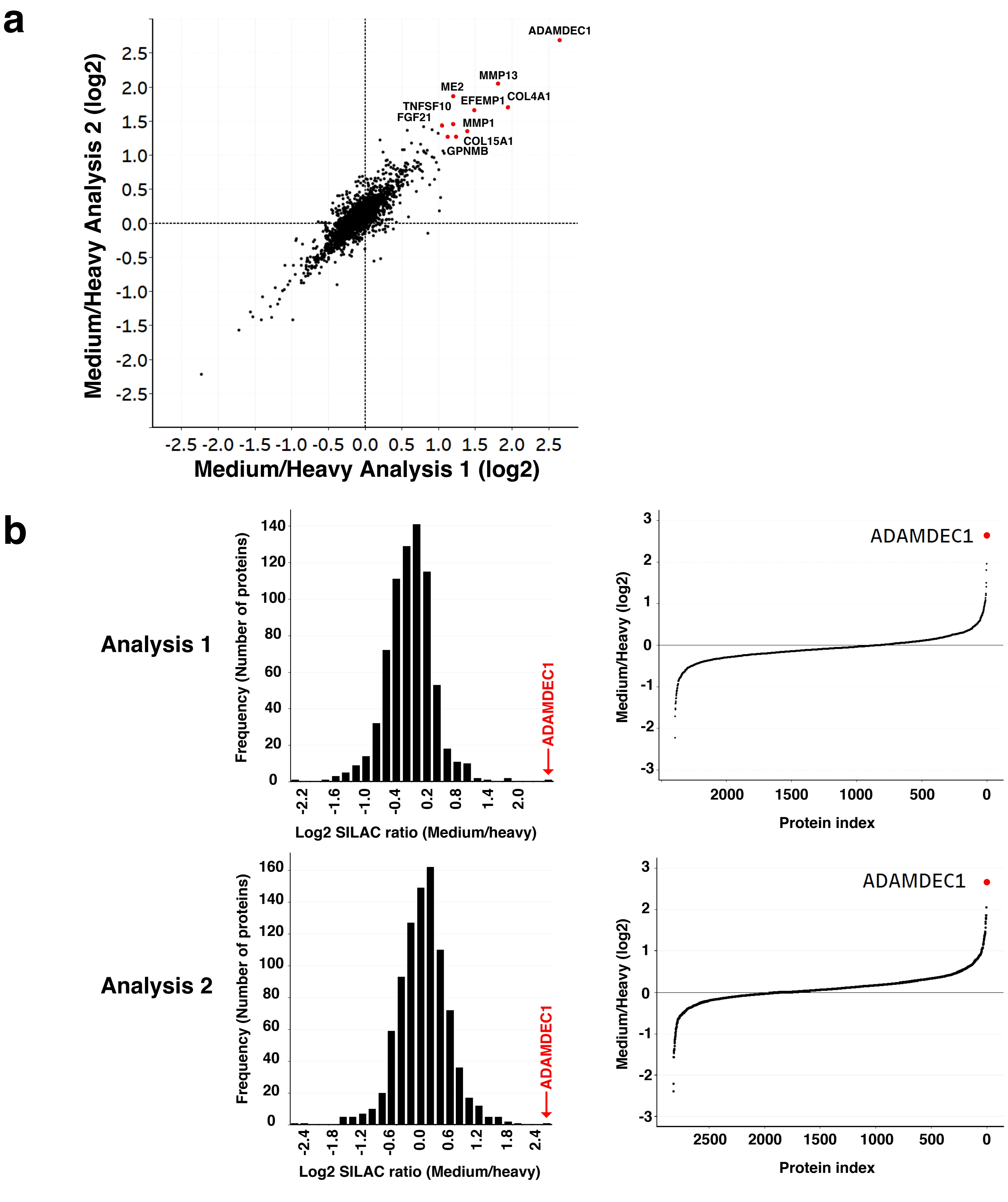

Figure S1. Scatter plot and histogram of Medium/Heavy ratios from two SILAC analyses. (**a**) From two SILAC analyses, Medium/Heavy ratio was determined for 2,272 proteins. Log2-transformed M:H SILAC ratios from two independent analyses are shown. Red dots denote top ten proteins. (**b**) Ratio distribution of all quantified proteins in two SILAC analyses. The M:H ratio of ADAMDEC1 was highest in both two analyses. Note that the total average of Log2 SILAC ratio of all proteins is around zero in both analyses.

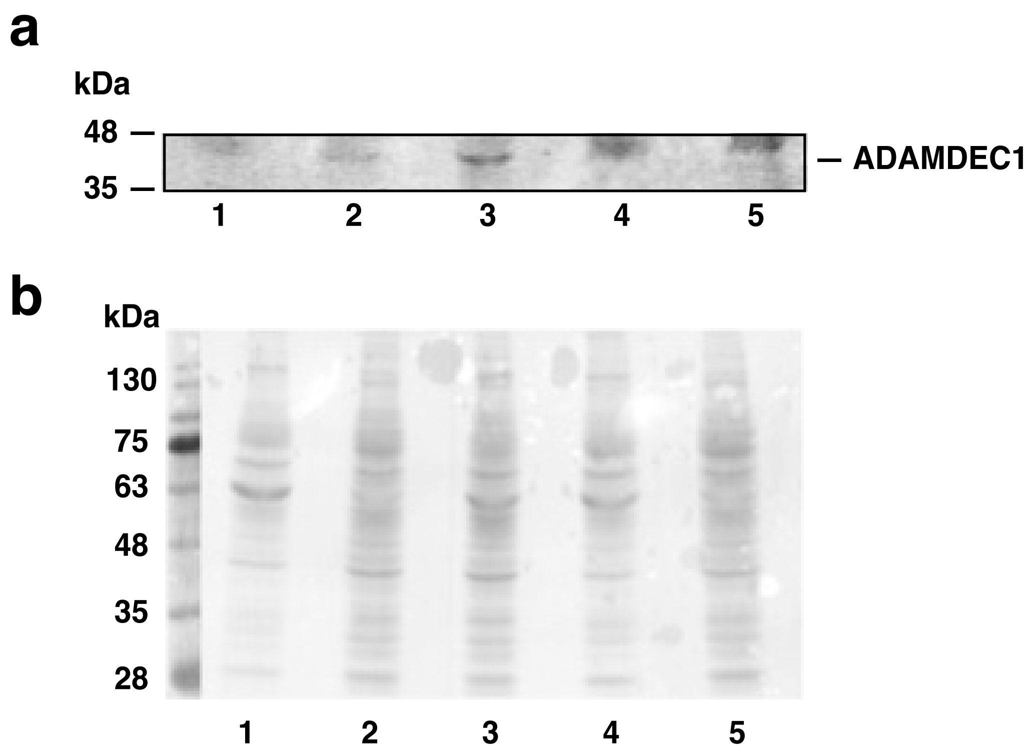

1 MDCK alone
2 RasV12 alone
3 MDCK:RasV12=1:1
4 MDCK ADAMDEC1-sh1 alone
5 MDCK ADAMDEC1-sh1:RasV12=1:1

Figure S2. Analysis of ADAMDEC1 expression in conditioned media by western blotting. Conditioned media from the indicated culture conditions were examined by western blotting with anti-human ADAMDEC1 antibody (**a**) and Coomassie Brilliant Blue protein staining (**b**).

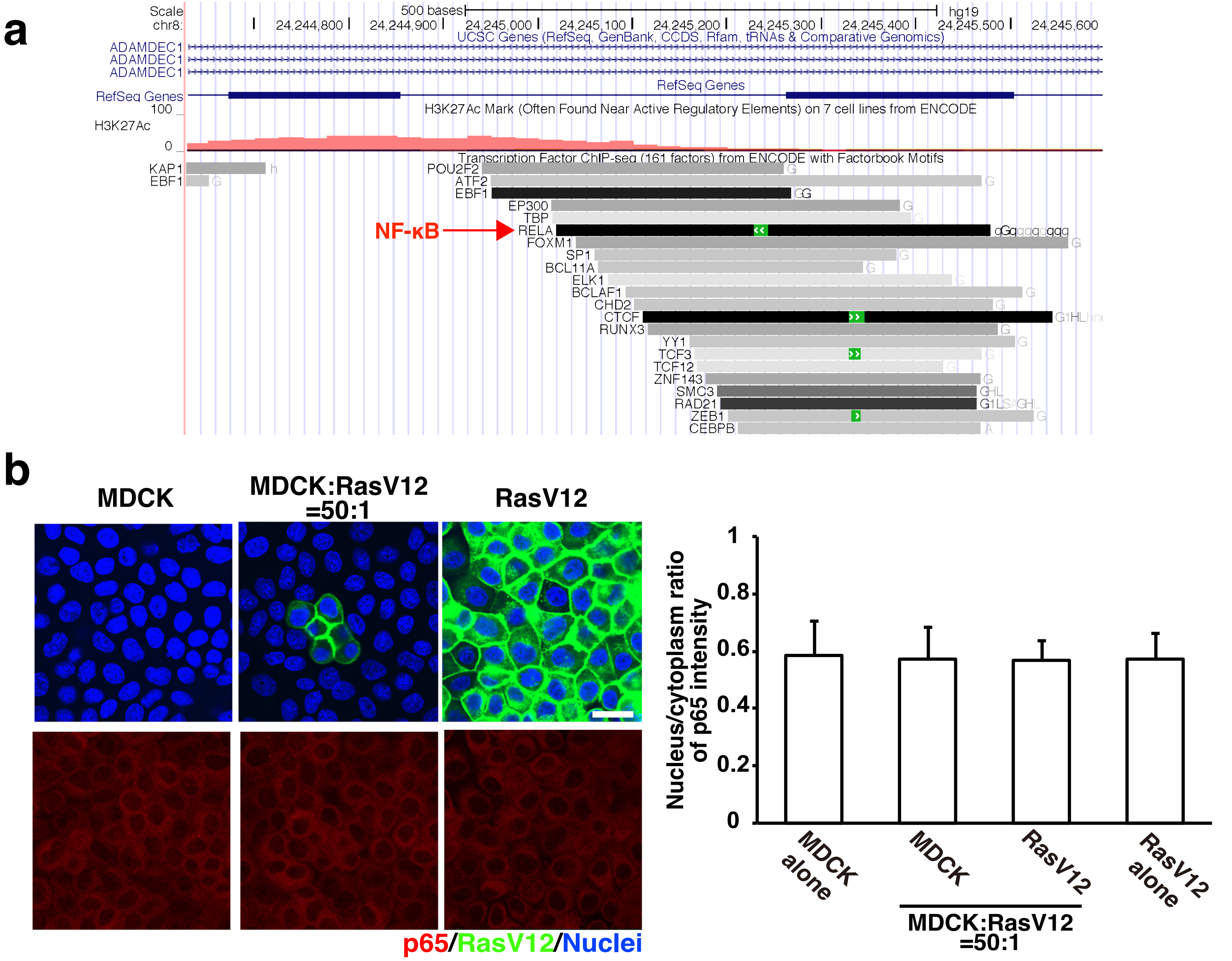

Figure S3. The NF-κB pathway is not activated in normal epithelial cells neighboring RasV12-transformed cells. (**a**) Database analysis in the promoter regions of human ADAMDEC1 using UCSC Genome Browser. The arrow indicates the potential binding sequence for NF-κB/RelA. (**b**) (Left panels) Confocal microscopic immunofluorescence images of p65, a subunit of NF-κB. MDCK and MDCK-pTR GFP-RasV12 cells were co-cultured or cultured alone. Cells were fixed at 16 h after tetracycline addition and stained with anti-p65 antibody (red) and Hoechst (blue). Scale bars, 10 μm. (Right panels) Quantification of the nucleus/cytoplasm ratio of p65 immunofluorescence intensity. Data are mean ± SD from three independent experiments. Note that the nuclear localization of p65 is not elevated in normal cells that surround RasV12 cells.

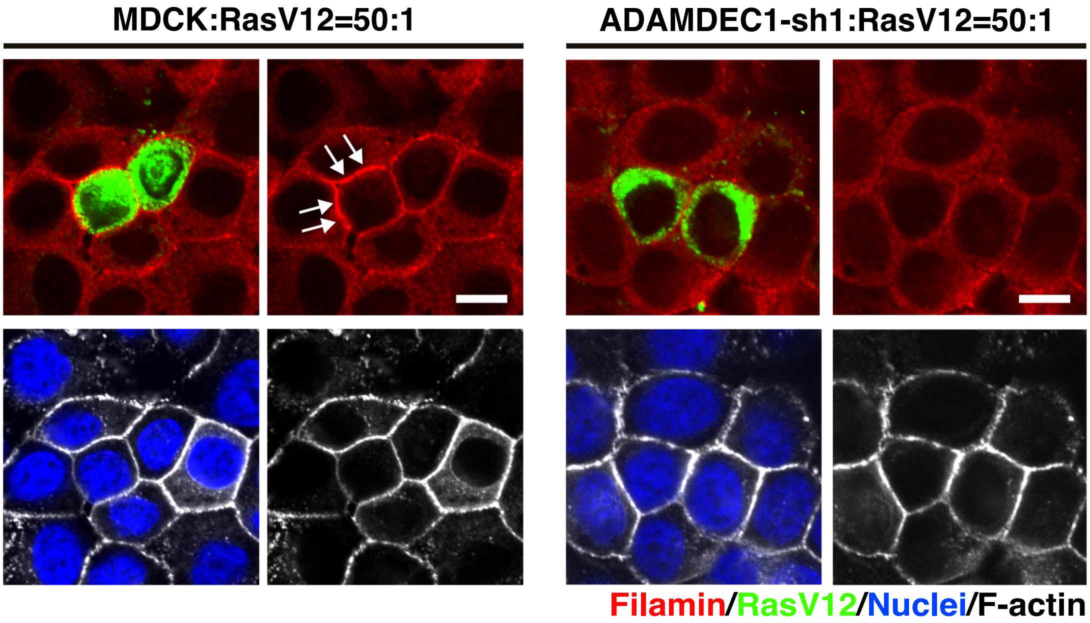

Figure S4. ADAMDEC1 knockdown in the surrounding normal cells suppresses filamin accumulation at the interface with RasV12-transformed cells. Confocal microscopic immunofluorescence images of filamin. MDCK-pTR GFP-RasV12 cells were co-cultured with MDCK or MDCK ADAMDEC1-shRNA1 cells. Cells were fixed at 16 h after tetracycline addition and stained with anti-filamin antibody (red), Alexa-Fluor-647-conjugated phalloidin (grey), and Hoechst (blue). Arrows indicate filamin accumulation. Scale bars, 10 µm.

Figure 2b)

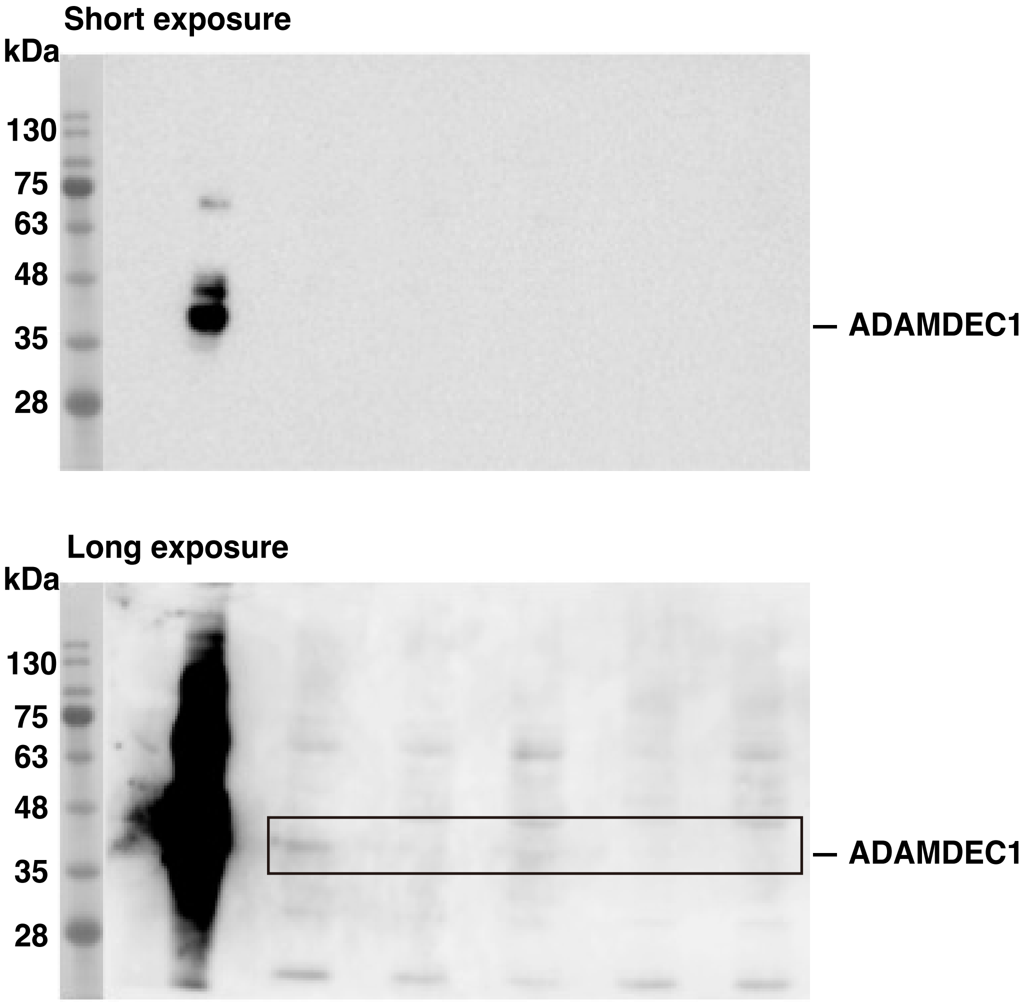

Figure S2a)

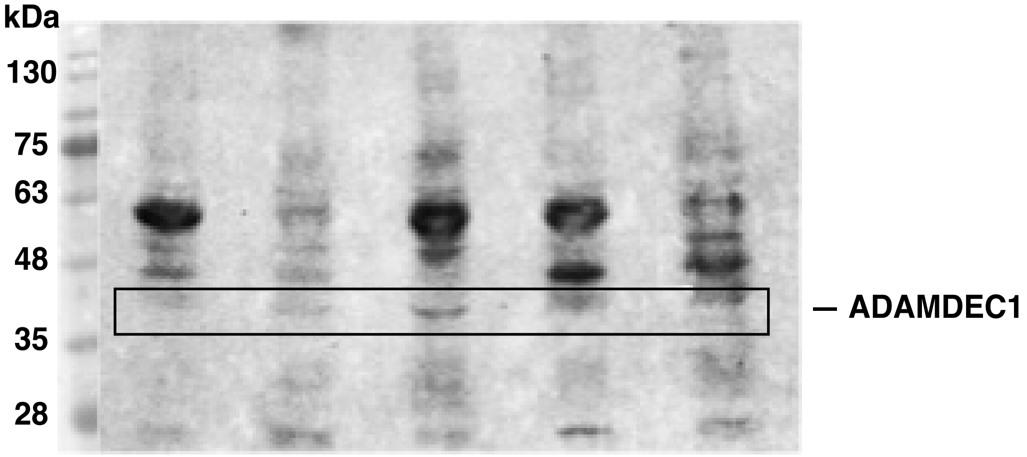

Figure S5. Full-length gels and blots.
